# Influence of aesthetic beauty models on body image in indigenous communities in Latin America: a systematic review

**DOI:** 10.3389/fpsyg.2026.1766017

**Published:** 2026-03-04

**Authors:** Juan Manuel Mancilla-Díaz, Mayaro Ortega-Luyando, Adriana Amaya-Hernández, Rosalía Vázquez-Arévalo, Luis Alberto Regalado-Ruiz, Rodrigo Erick Escartín-Pérez, Alejandro Pérez-Ortiz

**Affiliations:** Eating Disorders Laboratory, Facultad de Estudios Superiores Iztacala, Universidad Nacional Autónoma de México, Tlalnepantla, Mexico

**Keywords:** body image, eating disorder, indigenous world view, minoritized groups, model of aesthetic appreciation, sociocultural factors

## Abstract

**Introduction:**

Indigenous communities in Latin America remain underrepresented in body image research despite sociocultural transitions. This systematic review aimed to identify the influence of aesthetic beauty models and sociocultural factors on body image in Indigenous communities belonging to Latin America.

**Methods:**

Following PRISMA and SPIDER guidance, a systematic search (October 28, 2025) was conducted across five databases: PubMed, Web of Science, Scopus, Lilacs, and SciELO, as well as specialized eating-disorder journals. Observational and qualitative/mixed-methods studies involving Latin American Indigenous populations and addressing body image in relation to sociocultural/aesthetic models were included.

**Results:**

Sixteen studies met eligibility criteria, spanning multiple Indigenous and rural groups in Latin America. Across settings, findings indicated the coexistence of two partially competing frameworks: (1) persistence of local/traditional values in which larger bodies and/or curvilinearity may be associated with normality, health, status, or functionality, and (2) growing influence of Western/globalized appearance ideals emphasizing thinness and/or specific body proportions, particularly among younger women and in contexts of market integration and media access. Body dissatisfaction was frequent but heterogeneous in direction, with evidence of bidirectional dissatisfaction in some samples. Media effects were context-dependent: some studies supported causal influence of televisual exposure during technological transition, whereas others highlighted stronger roles for family, peers, and healthcare providers. Measurement concerns were recurrent, including limited cultural fit of silhouette-based tools.

**Conclusion:**

Body image in Latin American Indigenous communities reflects complex cultural negotiation between traditional meanings and Westernized ideals. More culturally grounded, longitudinal, and methodologically adapted research is needed to inform prevention and intervention approaches that protect body wellbeing without imposing Western frameworks.

## 1 Introduction

Body image, defined as the multidimensional mental representation that a person constructs about their own body, including perceptions, thoughts, feelings, and behaviors related to physical appearance, constitutes a central construct in understanding contemporary mental health and psychological wellbeing (Cash, 2004; Tylka and Wood-Barcalow, 2015). Over recent decades, research has consistently documented that culturally derived aesthetic models of beauty exert influence on body image and, by extension, on the development of eating disorders and body dissatisfaction (Abdoli et al., 2024; Lacroix et al., 2023).

The Tripartite Influence Model, proposed by Thompson et al. (1999), and still current today (Schaefer et al., 2021), has established that media, family, and peers constitute the primary socializing agents that transmit and reinforce dominant body aesthetic ideals, generating pressure to conform to specific beauty standards, particularly the thin ideal in women (Burke et al., 2021; Öztunç et al., 2025; Pérez-Bustinzar et al., 2023). This sociocultural influence operates fundamentally through two psychological mechanisms: internalization of the aesthetic ideal and social comparison with prevailing beauty standards. Nevertheless, the majority of empirical evidence supporting these theoretical models comes from studies conducted in populations from North America, Europe, and Oceania, predominantly in urban contexts and with participants from middle and upper socioeconomic strata (Harris et al., 2025; Iannattone et al., 2025; Kakar et al., 2023; Tennfjord et al., 2025).

This geographic and demographic concentration of research has generated significant gaps in knowledge about how sociocultural factors interact with body image in populations residing outside these predominant contexts. In response to this limitation, an increasing number of cross-cultural studies—including research conducted in non-Western and Latin American settings—has sought to expand the empirical base, thereby offering a more comprehensive understanding of body image–related phenomena across diverse cultural contexts (Odinga and Kasten, 2020; Silva and Cohen, 2025). However, this emerging literature remains limited in scope and coverage.

Notably, indigenous communities in Latin America and the Caribbean continue to be systematically underrepresented in the scientific literature on body image, despite comprising more than 800 indigenous peoples and approximately 58 million individuals (United Nations Educational Scientific Cultural Organization, 2025). This lack of information is particularly concerning, as indigenous populations present highly specific social, economic, and health vulnerabilities, such as socioeconomic marginalization, and simultaneous exposure to traditional values and Western cultural models (Mikhail and Klump, 2021; Williams-Ridgway et al., 2025).

The scarce studies that have explored body image in indigenous populations from other continents, particularly in Australia, New Zealand, Canada, and the United States, have documented findings that challenge traditional theoretical models, showing patterns of body dissatisfaction, preferences for bigger body sizes, and cultural protective factors that differ significantly from urban Western populations (Burt et al., 2020; Cleland et al., 2023). However, these findings may not be directly applicable to Indigenous communities in Latin America, given distinct historical trajectories, colonization processes, levels of market integration, and cultural configurations (Gone and Kirmayer, 2020; Gracey and King, 2009). In Latin American contexts, sociocultural dynamics such as colourism and skin-tone ideologies have been associated with body-related shame and appearance pressures (Teran et al., 2024). Additionally, changes in diet and nutrition practices associated with globalization, as well as forced displacement and migration, have been linked to nutrition-related health concerns and evolving social norms surrounding body and appearance (Castro-Prieto et al., 2024; United Nations Educational Scientific Cultural Organization, 2025).

Together, these findings underscore the importance of examining sociocultural dynamics such as colourism, displacement, and dietary change when considering body image and related constructs in underrepresented Indigenous populations in Latin America, despite the scarcity of direct empirical studies.

In Latin America, indigenous communities are experiencing significant cultural changes, where their traditions coexist with a growing influence of Western aesthetic models that arrive through urbanization, schooling, and mass media. This situation raises questions about how dominant aesthetic beauty models—centered on thinness, youth, and European physical features—interact with traditional cultural values that have historically assigned different meanings to the body, frequently associating bigger body size with health, prosperity, and social status (Martin and Latner, 2025; Odinga and Kasten, 2020).

Understanding the dynamics of Western aesthetic models in indigenous communities will not only contribute theoretical knowledge enabling the development of more inclusive and culturally sensitive models of body image, but also the design of prevention and treatment strategies for eating disorders and body image disorders that are culturally relevant and respectful of ethnic and cultural diversity. Furthermore, this knowledge contributes to making visible the experiences of historically marginalized groups in psychological and biomedical research, promoting more equitable and representative science.

Considering the arguments previously presented, the objective of the present research was to identify the influence of aesthetic beauty models and sociocultural factors on body image in indigenous communities belonging to Latin America.

## 2 Method

### 2.1 Search strategy

Based on the Preferred Reporting Items for Systematic reviews and Meta-Analyses (PRISMA; Page et al., 2021) and Sample, Phenomenon of Interest, Design, Evaluation, and Research type (SPIDER; Methley et al., 2014) methodologies, a systematic review was conducted on October 28, 2025. Regarding the SPIDER methodology, its purpose is the identification of qualitative or mixed-design studies (Methley et al., 2014).

### 2.2 Sources consulted for the review

The article search was conducted in five databases: PubMed, Web of Science, Scopus, Lilacs, and SciELO; as well as in seven specialized journals: *European Eating Disorders Review*; *International Journal of Eating Disorders*; *Journal of Eating Disorders*; *Eating and Weight Disorders-Studies on Anorexia, Bulimia and Obesity*; *Eating Disorders*; *Body Image*; and *Mexican Journal of Eating Disorders*.

### 2.3 Procedure for article selection

MeSH terms were selected from the PubMed Thesaurus (https://www.ncbi.nlm.nih.gov/mesh/). Table 1 presents the MeSH terms, keywords, and characteristics of articles to be identified, adjusted to the SPIDER methodology.

**Table 1:** MeSH terms, keywords, and characteristics of articles to be identified, adjusted to the SPIDER methodology.

**Element**	**Mesh or keyword/characteristic**
Sample	Indigenous peoples (MeSH Terms) and Latin America (MeSH Terms)
Phenomenon of Interest	(Aesthetic Appreciation Model or Aesthetic Beauty Model [Keyword]) and Body Image (MeSH Terms)
Design	Experimental or non-experimental
Evaluation	(Descriptive or comparative measures, prevalence, incidence, and regression models) or (experiences or perceptions)
Research type	Quantitative or qualitative or mixed design

The database filters were not used to identify the greatest number of articles. In the case of the Lilacs database, the search was conducted in specific sections of the articles, because the database did not allow searching in all elements, as in the other databases; consequently, the option containing Title, Abstract, and Subject Area was selected. Boolean operators [AND] and [OR] were employed to perform multiple combinations between MeSH terms and keywords.

The article search in the databases was conducted by JMMD, RVA, and LARR, while, to reduce the risk of bias in article selection, double-blind screening was performed by AAM and APO on the Rayyan platform (Ouzzani et al., 2016). In case of disagreement between the two authors, MOL re-evaluated the article considering the selection criteria.

### 2.4 Criteria for article selection

Prior to undertaking the search, the following inclusion criteria were established: (a) Design: Observational (cross-sectional, cohort studies, case-control, or qualitative); (b) Population: Being part of an indigenous people (indigenous community), belonging to a Latin American country, of any sex or age; (c) Search period: Published at any year; (d) Phenomenon of interest: Evaluating the interaction of aesthetic beauty models on body image; (e) Language: Articles published in Spanish or English. As part of the exclusion criteria, the following categories were considered: (a) Population: Not being part of an indigenous people or not considering a percentage of the sample belonging to an indigenous people; furthermore, not conducting the study in a Latin American country; (b) Types of studies: Systematic reviews, books, book chapters, and conference proceedings; (c) Language: Articles published in a language other than Spanish or English.

### 2.5 Analysis of articles

Information from the articles was retrieved according to what the PRISMA statement establishes (Page et al., 2021), such as: design, sample size, characteristics of the study sample, results, and risk of bias. To deepen the results, the country of origin of the research was included, as well as the instruments used (Sánchez-Sosa, 2004), the type of research and evaluation conducted (Methley et al., 2014).

## 3 Results

The search was initiated employing all MeSH terms and keywords, as well as their respective synonyms identified in the literature and the Boolean operators [AND] and [OR]; however, both in the databases and in the specialized journals, no results were found. Consequently, and after exploring multiple combinations, results were identified with the search strategy (Indigenous Peoples [All Fields]) AND (Body Image [All Fields]), that is, only with two keywords and identifying these words in any part of the article (All Fields). Table 2 shows all search strategies.

**Table 2:** Search strategy in databases and in specialized reviews of eating disorders.

**Database or journal**	**Search strategy (combinations among MeSH terms, keywords and paper sections)**	**Results**
Pubmed	((“Aesthetic” [All Fields] AND “Appreciation” [All Fields] AND “Model” [All Fields]) OR (“Aesthetic” [All Fields] AND “Beauty” [All Fields] AND “Model” [All Fields])) AND “indigenous peoples” [MeSH Terms] AND “body image” [MeSH Terms] AND “Latin America” [MeSH Terms]	0
	(((Aesthetic Appreciation Model OR aesthetic beauty model) AND (Indigenous Peoples)) AND (Body Image)) AND (Latin America)))	0
	(Indigenous Peoples[MeSH Terms]) AND (Body Image[MeSH Terms])	44
	(Indigenous Peoples) AND (Body Image)^*^	56
Web of science	(((Aesthetic Appreciation Model OR aesthetic beauty model [All Fields]) AND (Indigenous Peoples [All Fields])) AND (Body Image [All Fields])) AND (Latin America [All Fields])))	0
	(Indigenous Peoples [All Fields]) AND (Body Image [All Fields])^*^	76
Scopus	(Aesthetic Appreciation Model OR Aesthetic Beauty Model [Article Title, Abstract, Keywords]) AND (Indigenous Peoples [Article Title, Abstract, Keywords]) AND (Body Image [Article Title, Abstract, Keywords]) AND (Latin America [Article Title, Abstract, Keywords])	0
	(Indigenous Peoples [Article Title, Abstract, Keywords]) AND (Body Image [Article Title, Abstract, Keywords])	123
	(Aesthetic Appreciation Model OR Aesthetic Beauty Model [All fields]) AND (Indigenous Peoples [All fields]) AND (Body Image [All fields]) AND (Latin America [All fields])^*^	336
Lilacs	(Aesthetic Appreciation Model OR Aesthetic Beauty Model [Title, Abstract, Subject]) AND (Indigenous Peoples [Title, Abstract, Subject]) AND (Body Image [Title, Abstract, Subject]) AND (Latin America [Title, Abstract, Subject])	0
	(Indigenous Peoples [Title, Abstract, Subject]) AND (Body Image[Title, Abstract, Subject])^*^	7
SciELO	imagen corporal AND indígena AND Latinoamérica	0
	imagen corporal AND Latinoamérica^*^	3
European Eating Disorders Review	(Indigenous Peoples) AND (Body Image) AND (latin america)^*^	1
International Journal of Eating Disorders	(Indigenous Peoples) AND (Body Image) AND (latin america)^*^	6
Journal of Eating Disorders	(Indigenous Peoples) AND (Body Image) AND (latin america)^*^	3
Eating and Weight Disorders-Studies on Anorexia, Bulimia and Obesity	(Indigenous Peoples) AND (Body Image) AND (latin america)^*^	0
Eating Disorders	(Indigenous Peoples) AND (Body Image) AND (latin america)^*^	0
Body Image	(Indigenous Peoples) AND (Body Image) AND (latin america)^*^	22
Mexican Journal of Eating Disorders	Imagen corporal^*^	4

^*^Search strategy selected.

**Figure 1:**
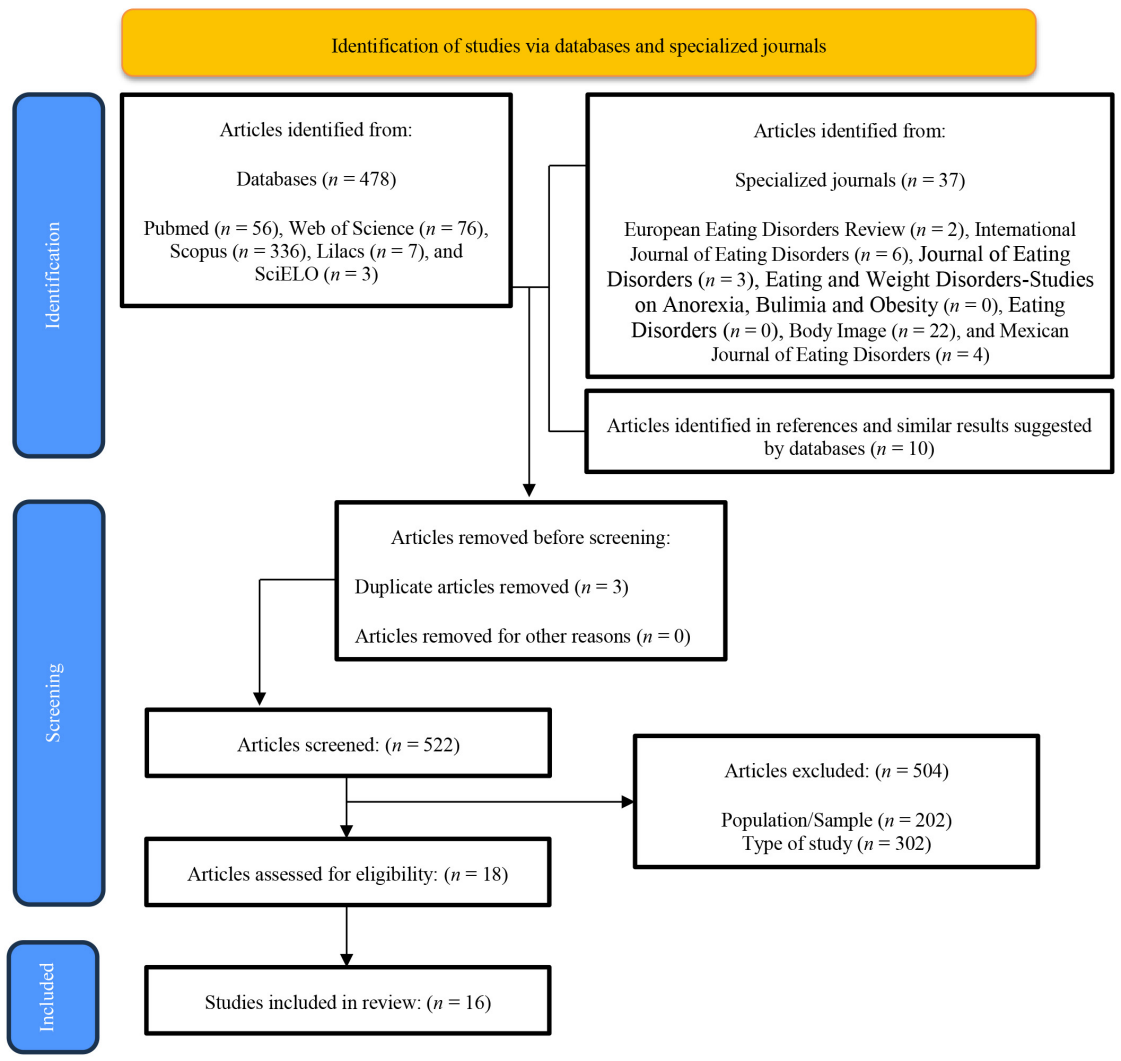
PRISMA flowchart studies selection.

In total, 478 articles were found in the databases, 37 in three specialized journals, and 6 in the references and in similar results suggested by the databases, yielding a total of 521 results; only 16 articles met the eligibility criteria. The flowchart of article selection can be observed in Figure 1.

### 3.1 General characteristics of the studies analyzed

Sixteen studies met the established inclusion criteria. The evidence base comprised research conducted in Latin America (Mexico, Argentina, Chile, Brazil, Nicaragua, Guatemala, and Bolivia) and, in some cases, cross-cultural comparisons including non-Latin American settings.

Methodologically, the included studies were heterogeneous, encompassing cross-sectional quantitative designs, mixed-methods investigations, qualitative studies (e.g., focus groups), and studies with longitudinal and/or experimental components in contexts of sociocultural transition (e.g., media exposure).

### 3.2 Characteristics of included studies

The 16 studies cover Indigenous and/or rural populations from Mexico, Argentina, Chile, Brazil, Nicaragua, Guatemala, and Bolivia, and some include cross-cultural comparisons (e.g., with the United Kingdom and Uganda) to contrast sociocultural mechanisms. Study designs were heterogeneous: quantitative, cross-sectional, mixed-methods (quantitative-qualitative), qualitative (focus group/ethnographic), studies with longitudinal components in Nicaragua, and “natural experiments” focused on media exposure.

Sample sizes ranged from small qualitative samples to larger community-based surveys. The populations represented included Mayas (Mexico), Qom (Argentina), Mapuche (Chile), Khisêdjê (Brazil), indigenous women from Hidalgo and Oaxaca (Mexico), and ethnically diverse rural communities in Nicaragua, as well as additional indigenous/rural groups examined in Guatemala and Bolivia (Andres et al., 2026; Boothroyd et al., 2016, 2020; Daiy et al., 2020, 2023; Guzmán-Saldaña et al., 2017; Maupin and Brewis, 2014; Odinga and Kasten, 2020; Pérez and Estrella, 2014; Pérez and Romero, 2008; Rosas et al., 2015; Santos et al., 2020; Sorokowski et al., 2014; Thornborrow et al., 2020, 2022, 2025).

### 3.3 Assessment instruments used

Across the 16 included studies, body image was assessed using a range of methods. Figure rating approaches (commonly Stunkard-type silhouettes or adaptations) were frequently used to measure perceived current body, ideal body, and/or the body considered healthiest, often alongside anthropometric assessment (e.g., Body Mass Index [BMI], in some cases waist circumference and waist to hip ratio; Daiy et al., 2020, 2023; Odinga and Kasten, 2020; Pérez and Estrella, 2014; Pérez and Romero, 2008; Santos et al., 2020; Sorokowski et al., 2014). Eating-disorder risk and body-shape concerns were assessed in some student/community samples using standardized questionnaires such as the Eating Attitudes Test and the Body Shape Questionnaire (Rosas et al., 2015), while sociocultural internalization and pressures from family/peers were measured in several Nicaraguan studies examining sociocultural pathways to risk (Andres et al., 2026; Thornborrow et al., 2022). Importantly, some studies used culturally adaptive visual methods (e.g., 3D avatar-based approaches) to capture locally salient ideals of body shape and proportions rather than size alone, or considered the effect of content displayed on television on body size preference (Boothroyd et al., 2020; Thornborrow et al., 2022). Multiple studies also noted limitations in cross-cultural measurement equivalence, particularly regarding the cultural fit of silhouette stimuli for indigenous phenotypes and norms (Guzmán-Saldaña et al., 2017; Santos et al., 2020).

### 3.4 Nutritional and anthropometric profile

A frequent background finding—especially in community-based Indigenous samples—was the high prevalence of excess weight, which contextualizes the formation of body ideals and dissatisfaction.

In Mayan communities in Yucatán (Mexico), total overweight/obesity was 70% in Mucuyché and 53% in Xanláh (Pérez and Estrella, 2014). In the Qom community (Argentina), excess weight was also high: 77% of women and 76% of men had overweight/obesity in the larger sample (*n* = 273); in a later study, mean BMI was ≈29.7 kg/m^2^ (Daiy et al., 2020, 2023).

In the Khisêdjê population (Brazil), overweight was ≈42% and obesity ≈5.3%, with higher prevalence in men (Santos et al., 2020). Among Indigenous women from Hidalgo (Mexico), 40.1% obesity and 18% overweight were observed (Guzmán-Saldaña et al., 2017). In Oaxaca (Mexico), women from the coast showed higher excess weight (e.g., 28% overweight and 14% obesity) than women from the highlands (24% overweight and 7% obesity), along with differences in ideals and body self-evaluation (Pérez and Romero, 2008).

### 3.5 Body image outcomes: ideals, satisfaction and dissatisfaction

Overall, the studies show the coexistence of: (1) traditional/local values that may normalize or positively value bigger or curvier bodies, and (2) a growing influence of Western/globalized ideals (thinness and/or body proportions), especially among younger people and in contexts with greater market integration, urbanization, or media exposure.

#### 3.5.1 Persistence of local/traditional valuations

In Mayan communities (Guatemala and Yucatán), “being chubby” is described as part of cultural normality and a marker of status/prosperity; additionally, food choices were linked to prestige and social recognition rather than nutritional value (Maupin and Brewis, 2014; Pérez and Estrella, 2014). In Khisêdjê (Brazil), 61.8% reported satisfaction with their body image; most chose mid-range silhouettes as current and ideal body, although concordance between silhouettes and BMI was low (Santos et al., 2020).

In rural women from Nicaragua's Caribbean coast (qualitative), local ideals were described as relatively less rigid and more oriented toward curvilinearity and general appearance aspects; many women reported body acceptance (“God-given bodies”), with nuances by ethnic group (Creole women showing greater self-confidence; Mestizo women discussing dieting more; Thornborrow et al., 2025). In Guatemala, Q'eqchi' women showed preference for bigger bodies and higher body self-esteem compared with women from Western urban contexts (Odinga and Kasten, 2020). Among Tsimane' men (Bolivia), a preference for a higher ideal BMI (≈26.1) and an association between isolation and preference for larger bodies were observed (Sorokowski et al., 2014).

#### 3.5.2 Western/globalized influence and shifting norms

Western influence appears more clearly in communities with greater external exposure or media access. In Mucuyché (Yucatán), greater pressure toward thinner bodies was observed: participants perceived themselves as overweight, avoided identifying with obese silhouettes, and desired thinner figures (Pérez and Estrella, 2014).

In the Qom community (Argentina), a transitional pattern is documented: among women—especially older women—idealization of overweight as “healthy” persists, but younger women select thinner silhouettes as healthier, suggesting generational change (Daiy et al., 2020). A later mixed methods study also reported a temporal shift toward thinner “healthy” silhouettes when comparing 2010 vs. 2019 data (Daiy et al., 2023).

In Nicaragua, “natural experiment” studies and experimental designs indicate that television consumption/exposure is associated with thinner ideals: for example, communities with greater TV access preferred lower ideal BMI than communities without TV, and brief exposure to thin models recalibrated aesthetic norms (Boothroyd et al., 2020). Additionally, some findings suggest that media effects may manifest more strongly in shape/proportion (e.g., ideal waist-to-hip ratio (WHR)/waist-to-bust ratio WBR) than in “size” (ideal BMI) among rural women in technological transition (Thornborrow et al., 2020).

### 3.6 Eating-disorder risk and body dissatisfaction correlates

Body dissatisfaction was high in several samples, although it did not always point exclusively toward thinness, reflecting coexisting ideals.

Among Indigenous women from Hidalgo, 83.2% reported body dissatisfaction in two directions: 51.5% wanted to be thinner and 31.7% wanted to be more robust (Guzmán-Saldaña et al., 2017). Among students in Chile, there were no significant differences between Mapuche and non-Mapuche in eating-disorder risk or body-shape concern; gender (women) and excess weight were associated with higher body image concern, and the BMI–body concern correlation was moderate (*r* ≈ 0.38; Rosas et al., 2015).

In women from different communities in Nicaragua, internalization of beauty standards led to greater body dissatisfaction and increased eating-risk/symptoms, while body appreciation functioned as a protective factor; additionally, the risk of eating-disorder increased longitudinally in follow-up (Thornborrow et al., 2022).

### 3.7 Sociocultural drivers: media vs. proximal influences (family/peers/health providers)

The studies suggest that sociocultural influence does not depend exclusively on media. In Qom (Argentina), media/technology exposure was not robustly associated with ideals/dissatisfaction in quantitative analyses; however, interviews emphasized greater influence of peers, family, and health providers than media, alongside persistent cultural values of body acceptance (Daiy et al., 2023). In contrast, in Nicaragua, evidence from natural experiments and brief exposure to media stimuli supports that visual media can recalibrate body norms (toward thinner ideals) in populations undergoing transition (Thornborrow et al., 2022).

### 3.8 Gender and generational patterns

Sex- and generation-related patterns appear, although they vary by population and construct. In Qom, the pattern was markedly “gendered and generational”: older women maintained bigger body ideals, while younger women shifted toward thinner body ideals; the same generational pattern was not observed as strongly in men (Daiy et al., 2020). In Chile, female sex was associated with higher eating-disorder risk and greater body image concern, regardless of Mapuche/non-Mapuche status (Rosas et al., 2015).

In male-focused studies (transcultural comparisons), the focus shifted from thinness to muscularity: peer pressure and sociocultural internalization predicted drive for muscularity, with cross-country differences (e.g., higher drive for muscularity in the UK and higher body appreciation in Nicaragua; Thornborrow et al., 2020).

### 3.9 Methodological strengths and challenges of the analyzed studies

#### 3.9.1 Strengths

Overall, the reviewed studies show methodological diversity, which strengthens understanding of the topic by triangulating quantitative, qualitative, and—in some cases—longitudinal/experimental evidence. A key strength is the inclusion of objective measures (weight, height, BMI, and occasionally waist circumference), which allows researchers to interpret perceived ideals and body satisfaction against an anthropometric background (e.g., in Maya, Qom, and Khisêdjê samples). In addition, several studies used mixed-methods designs (questionnaires plus interviews), enhancing interpretive validity by situating body-image constructs within local cultural meanings—for example, how body size is linked to status, health, or food practices, and how the relative influence of media compares to that of proximal agents such as family, peers, and health providers.

Another methodological strength is that some studies go beyond purely descriptive associations and contribute evidence on plausible mechanisms. In Nicaragua, the presence of longitudinal components and natural experiment/experimental approaches related to television exposure increases causal plausibility regarding how media may recalibrate body ideals and norms in communities undergoing sociocultural transition. Likewise, the use of more culturally flexible measurement approaches—such as 3D avatars to capture ideals of shape/proportions (not only “size”)—is an important methodological innovation that can reduce bias from Western visual stimuli and improve sensitivity to locally salient features (e.g., curves, waist, proportions).

#### 3.9.2 Challenges

The most frequently identified challenge included small or unbalanced sample sizes, particularly in the qualitative components and in comparative studies where one group was considerably smaller than the other. Several studies employed convenience or non-probabilistic sampling, which limits generalization of results.

Another challenge was the low applicability of the Stunkard Figure Rating Scale in some indigenous populations. Santos et al. (2020) documented low concordance between selected silhouettes and measured BMI, suggesting that silhouettes may not be culturally appropriate for indigenous populations. Likewise, Guzmán-Saldaña et al. (2017) noted that the silhouettes used did not correspond to the physical features of rural indigenous women, particularly those of short height (average height of 1.46 meters).

Language barriers constituted another challenge to consider. Guzmán-Saldaña et al. (2017) acknowledged that researchers did not speak indigenous languages and had to use trained interpreters, which could potentially affect communication accuracy. Daiy et al. (2023) noted that interviewers were outsiders and non-indigenous, potentially influencing participants' responses.

The predominant cross-sectional designs make it impossible to establish causal relationships between the studied variables. Daiy et al. (2020) acknowledged that their design did not allow distinguishing cohort effects vs. age effects, limiting the interpretation of the observed generational differences.

Finally, several authors acknowledged the scarcity of previous research on the topic in rural and indigenous populations, making it difficult to compare and contextualize the results obtained.

## 4 Discussion

The aim of this systematic review was to identify the influence of aesthetic beauty models and sociocultural factors on body image in indigenous communities belonging to Latin America. The findings of this systematic review reveal a scarcity of studies focused on Latin American indigenous populations, with only 16 articles that met the inclusion criteria, which evidences very limited regional representation considering the diversity of more than 800 indigenous peoples in Latin America (United Nations Children's Fund, 2023).

Across the 16 included studies, body image in Latin American indigenous and rural communities was not characterized by a single dominant aesthetic model, but rather by the coexistence of two partially competing frameworks: (1) the persistence of local/traditional values in which bigger bodies and/or curvilinearity may be associated with normality, health, prosperity, fertility, or functional strength; and (2) increasing exposure to Western/globalized beauty ideals emphasizing thinness and/or particular body proportions, especially among younger generations and in contexts of market integration and media access (Boothroyd et al., 2020; Daiy et al., 2020, 2023; Pérez and Estrella, 2014; Thornborrow et al., 2022).

This duality is particularly evident when comparing communities or subgroups with different degrees of external exposure. For example, in Mayan communities (Guatemala and Yucatán), the community described as more exposed to external influences showed stronger pressure toward thinner bodies and a tendency to avoid identifying with obese silhouettes, whereas the less exposed community maintained stronger cultural normalization of “being chubby” as status and prosperity (Maupin and Brewis, 2014; Pérez and Estrella, 2014). Similarly, among Qom participants, generational differences (especially among women) suggest that younger women are adopting thinner “healthy” ideals compared to older women, consistent with an acculturative shift that may unfold unevenly across gender and cohort (Daiy et al., 2020, 2023).

These findings align with broader Indigenous body-image research outside Latin America showing that dominant societal beauty ideals often reflect colonial histories and exclude Indigenous bodies. In Australia, Indigenous women described racism and colourism as negatively affecting body image, while developing a strong cultural identity mitigated these impacts by facilitating belonging and body acceptance—highlighting cultural identity as a protective factor (Chalmers et al., 2022).

A central contribution from the Nicaraguan evidence base is that sociocultural exposure may shift ideals not only toward lower weight but also toward specific body shapes and proportions. Thornborrow et al. (2022), found that components of ideal body shape (curvaceousness) were associated with film/television consumption, whereas body mass itself was not necessarily the primary axis of change. Similarly, Boothroyd et al. (2020) provided convergent cross-sectional, longitudinal, and experimental evidence that media exposure can directly influence body size ideals in a population undergoing technological transition.

This is important because much of the classic body-image literature conceptualizes Westernization primarily as “thin-ideal internalization.” The Latin American indigenous/rural evidence suggests the need for a more nuanced framework that distinguishes size-based ideals (body mass) from shape-based ideals (waist-to-hip or other proportionality cues), particularly in contexts where curvilinearity is culturally salient and thinness is not the only aspirational endpoint (Boothroyd et al., 2020; Sorokowski et al., 2014; Thornborrow et al., 2022).

Complementarily, recent scholarship argues that Indigenous knowledge systems provide a robust foundation for positive body image via body functionality—valuing what the body can do rather than how it looks. Martin and Latner propose that Indigenous cultural practices and values foster appreciation for embodied lived experience, and that the relationship between an Indigenous body and land, culture, spirituality, and community promote a holistic celebration of functional capabilities (Martin and Latner, 2025). This perspective resonates strongly with several included Latin American findings in which larger body size may be valued for social meaning and/or daily functionality rather than aesthetic appearance alone (Pérez and Estrella, 2014; Pérez and Romero, 2008; Santos et al., 2020).

A key synthesis from the included literature is that “sociocultural influence” is not reducible to media exposure. In the Qom mixed-methods study, quantitative analyses did not find strong direct associations between media/technology exposure and ideals, healthy-body norms, or body dissatisfaction, while qualitative interviews suggested stronger influence from family, peers, and health providers and the persistence of culturally grounded acceptance (Daiy et al., 2023). In contrast, in Nicaragua, media exposure has been observed “in action,” with experimental and longitudinal components supporting a causal role of television in recalibrating ideals (Boothroyd et al., 2020; Thornborrow et al., 2022).

Rather than treating these findings as contradictory, they likely indicate that media effects are context-dependent and moderated by: (a) stage of technological transition (media-naïve vs. saturated), (b) local normative climate and cultural identity strength, and (c) the relative salience of proximal agents (family/peers/healthcare) and structural conditions (poverty, marginalization, food insecurity; Boothroyd et al., 2020; Daiy et al., 2023; Thornborrow et al., 2022).

Several indigenous/community-based samples in this review were characterized by high prevalence of overweight/obesity, yet dissatisfaction was not uniformly aligned with a thinness goal. For example, among indigenous women in Hidalgo, high dissatisfaction coexisted with desire to be thinner and desire to be more robust, underscoring heterogeneous ideals rather than a single Westernized standard (Guzmán-Saldaña et al., 2017). This heterogeneity suggests that interventions focused solely on thin-ideal internalization may overlook locally meaningful drivers of dissatisfaction, including health discourses, functionality constraints, stigma processes, and culturally specific social meanings attached to body size (Guzmán-Saldaña et al., 2017; Santos et al., 2020).

The distinction between “healthy body” and “ideal body” is also supported by evidence from Indigenous youth research in the United States. Rinderknecht and Smith used a modified and validated body-image measure for Native American youth and found that the median figure perceived as healthiest was moderate (4.0 on an 8-figure scale), while the figure most likely to develop diabetes was the largest (8.0), indicating that youth differentiated health risk from attractiveness and that many still desired to be thinner (41% of boys; 61% of girls; Rinderknecht and Smith, 2002). Although not Latin American, this study underscores the conceptual importance of distinguishing (a) aesthetic ideals, (b) health norms, and (c) culturally mediated meanings of weight in Indigenous populations.

Across the included studies, a major limitation concerns the cultural appropriateness of measurement tools, particularly silhouette scales developed in Western populations. Several studies reported low agreement between silhouette selection and BMI or noted phenotype mismatch (e.g., stature and body proportions), language barriers requiring interpreters, and potential reactivity to outsider interviewers (Guzmán-Saldaña et al., 2017; Santos et al., 2020). These issues can inflate apparent “misperception” or obscure true associations, and they complicate comparisons across groups.

The studies that introduced culturally flexible methods—such as 3D avatars that allow participants to construct their ideal without predefined Western stimuli—represent a promising direction for improving construct validity in Latin American Indigenous research (Boothroyd et al., 2020; Thornborrow et al., 2022).

## 5 Implications and future directions

Taken together, the evidence suggests that Latin American Indigenous and rural communities may experience a dual burden: increasing exposure to appearance-related pressures and internalization pathways associated with body dissatisfaction and eating-disorder risk, alongside persistent contexts of socioeconomic vulnerability and high excess weight prevalence. Prevention and health promotion should therefore prioritize culturally anchored protective factors (e.g., cultural identity, community belonging, and functionality-based body appreciation) while avoiding the uncritical transfer of Western thin-centric frameworks (Chalmers et al., 2022; Martin and Latner, 2025).

Future research should: (1) expand coverage across the diversity of Latin American Indigenous peoples, (2) employ longitudinal designs to disentangle cohort vs. age effects, (3) develop and validate culturally grounded instruments (including shape- and functionality-based measures), and (4) explicitly model multi-level sociocultural influences (media, peers, family, healthcare, and structural conditions) to clarify when and why Western beauty ideals become internalized or resisted.

## 6 Conclusions

This systematic review shows that in Indigenous and rural communities in Latin America, traditional body values (which may favor larger/curvier bodies) coexist with the growing influence of Western ideals, which is more evident among younger women and in contexts of greater integration and sociocultural exposure. Body dissatisfaction is neither uniform nor always oriented toward thinness, reflecting complex processes of cultural transition. More representative and longitudinal research, using culturally validated instruments, is needed to guide preventive interventions that promote body wellbeing without imposing Western frameworks or undermining culturally grounded protective factors.
